# IFNβ secreted by microglia mediates clearance of myelin debris in CNS autoimmunity

**DOI:** 10.1186/s40478-015-0192-4

**Published:** 2015-04-03

**Authors:** Magdalena Kocur, Reiner Schneider, Ann-Kathrin Pulm, Jens Bauer, Sonja Kropp, Michael Gliem, Jens Ingwersen, Norbert Goebels, Judith Alferink, Timour Prozorovski, Orhan Aktas, Stefanie Scheu

**Affiliations:** Institute of Medical Microbiology and Hospital Hygiene, Medical Faculty, University of Duesseldorf, Universitaetsstr. 1, 40225 Duesseldorf, Germany; Department of Neurology, Medical Faculty, University of Duesseldorf, Duesseldorf, Germany; Department of Psychiatry, University of Muenster, Muenster, Germany; Cluster of Excellence EXC 1003, Cells in Motion, Muenster, Germany

**Keywords:** IFNβ, Microglia, CNS autoimmunity, Multiple sclerosis, EAE

## Abstract

**Introduction:**

Multiple sclerosis (MS) is a chronic demyelinating disorder of the central nervous system (CNS) leading to progressive neurological disability. Interferon β (IFNβ) represents a standard treatment for relapsing-remitting MS and exogenous administration of IFNβ exhibits protective effects in experimentally induced CNS autoimmunity. Also, genetic deletion of IFNβ in mice leads to an aggravation of disease symptoms in the MS model of experimental autoimmune encephalomyelitis (EAE). However, neither the underlying mechanisms mediating the beneficial effects nor the cellular source of IFNβ have been fully elucidated.

**Results:**

In this report, a subpopulation of activated microglia was identified as the major producers of IFNβ in the CNS at the peak of EAE using an IFNβ-fluorescence reporter mouse model. These IFNβ expressing microglia specifically localized to active CNS lesions and were associated with myelin debris in demyelinated cerebellar organotypic slice cultures (OSCs). In response to IFNβ microglia showed an enhanced capacity to phagocytose myelin in vitro and up-regulated the expression of phagocytosis-associated genes. IFNβ treatment was further sufficient to stimulate association of microglia with myelin debris in OSCs. Moreover, IFNβ-producing microglia mediated an enhanced removal of myelin debris when co-transplanted onto demyelinated OSCs as compared to IFNβ non-producing microglia.

**Conclusions:**

These data identify activated microglia as the major producers of protective IFNβ at the peak of EAE and as orchestrators of IFNβ-induced clearance of myelin debris.

**Electronic supplementary material:**

The online version of this article (doi:10.1186/s40478-015-0192-4) contains supplementary material, which is available to authorized users.

## Introduction

Multiple sclerosis (MS) is an inflammatory demyelinating disease of the central nervous system (CNS). More than 20 years ago, interferon β (IFNβ) became the first immunomodulatory substance used in the treatment of MS. IFNβ is currently the most commonly used therapy for relapsing-remitting MS (RRMS), reducing relapses and severity of disease [1,2]. Genetic ablation of IFNβ or its receptor leads to an increased severity of experimental autoimmune encephalomyelitis (EAE) [3,4] a mouse model exhibiting clinical, neuropathological, and immunological disease manifestations of MS [5]. Conversely, induction of endogenous IFNβ by poly (I:C) stimulation suppresses EAE, confirming its protective role in CNS autoimmunity [6]. Today, neither the cellular source of type I IFN in EAE nor its localization relative to responding cells is known due to a lack of sufficiently sensitive *in situ* tools.

Also, the exact mechanisms underlying the protective effects of IFNβ remain incompletely understood. Various IFNβ-mediated modes of action have been suggested, including (i) downregulation of matrix metalloproteinase 9 thereby reducing tissue damage and inflammation, (ii) prevention of effector cell migration by downregulating the adhesion molecule very late antigen-4 (VLA-4) [7,8], (iii) downregulation of MHC II molecules on antigen presenting cells combined with upregulation of the inhibitory PD-L1 and PD-L2 ligands [9,10], (iv) inhibition of T cell proliferation [11], (v) the induction of immune cell apoptosis [12] and (vi) most recently the induction of FoxA1^+^ T regulatory cells [13]. Removal of myelin debris has been suggested as an essential protective mechanism ameliorating IFNγ-mediated neuroinflammation by downregulating the transcription levels of pro-inflammatory factors like TNF, IL-1β, or iNOS [14]. Reducing inflammation by enhancing phagocytosis efficacy may therefore represent a novel therapeutic approach in the treatment of neuroinflammation as observed in MS. Until now, however, a direct role for IFNβ in phagocytosis of myelin or axonal debris has not been shown and the functional consequences of microglial phagocytosis remain largely unexplored.

Though IFNβ was shown to delay disease progression, adverse side effects such as depression, flu-like symptoms, skin reactions, and bone marrow suppression have limited its use [15]. Also, IFNβ treatment is not always effective, as about 30% to 50% of patients experience breakthrough disease. One reason is production of neutralizing antibodies to IFNβ resulting in reduced or complete loss of therapeutic efficacy [16]. Moreover, there is the risk that a long term systemic “block” of inflammation could affect the elicitation of immune responses required for host defense. Therefore, it is of great interest to identify the cellular source and define the mechanisms associated with IFNβ-mediated protection against neuroinflammation as a first step in the development of cell-specific treatment regimes.

In this study we characterized the cell type responsible for IFNβ production and its impact on microglia effector functions in EAE using a yellow fluorescent protein (YFP) IFNβ reporter mouse model, organotypic slice cultures, and adult microglia cell cultures. We demonstrate that microglia are the primary IFNβ producing cells during the peak phase of EAE. We further show that IFNβ induces localization of microglia in close proximity to myelin debris and subsequently increases microglial phagocytotic activity. These findings and the fact that IFNβ-producing microglia orchestrated the clearance of myelin debris in organotypic cerebellar slice cultures reveal a so far unknown function of IFNβ. Our data further suggest that future IFNβ-based therapies targeting these cells in the CNS can be developed for treatment of demyelinating CNS disorders.

## Materials and methods

### Mice and EAE induction

Female C57BL/6 N mice were purchased from Charles River. IFNβ^mob/mob^ (*m*essenger *o*f IFN *b*eta: IFNβ/YFP reporter mouse) [17], IFNβ^−/−^ [18] and IFNAR1^−/−^ [19] mice were backcrossed for at least 10 generations onto C57BL/6 N background. PLP-EGFP mice were used for indicated organotypic slice culture experiments [20]. All mice were housed under specific pathogen free conditions in the animal research facility of the University of Duesseldorf. Mice at 6–12 weeks of age were used for all experiments. Active EAE was induced by tail-base immunization with 200 μg of MOG_35–55_ (Biotrend) in complete Freund's adjuvant (CFA), supplemented with Mycobacterium tuberculosis H37RA (10 mg/ml) (Difco Laboratories) and 200 ng pertussis toxin (Sigma) on 0d and 2d. A control group was treated with CFA only and 200 ng pertussis toxin on 0d and 2d. Classification of disease progression: 0 no paralysis; 0.5 partial paralysis of tail; 1.0 paralysis of tail; 1.5 unilateral paralysis of hind legs; 2.0 bilateral paralysis of hind legs; 2.5 bilateral paralysis of hind legs with unilateral weakness of forelegs; 3 tetraparesis (abort criteria); 4 death. All animal experiments were approved by the government of North Rhine-Westphalia (Az.: 84–02.04.2013.A466, Az.: 8.87-50.10.34.08.241).

### Organ and CNS mononuclear cell isolation

Mice were anesthetized and thereafter perfused with 50 ml ice cold PBS. For qRT PCR and flow cytometric analysis spleen, spinal cord and brain were removed. For flow cytometric analysis spinal cord and brain were homogenized and digested with collagenase/dispase (Roche) followed by DNase I (Roche) digestion. CNS derived mononuclear cells were isolated from the 30%/70% interface of a Percoll gradient after centrifugation at 800 × g for 25 min at room temperature. For RNA isolation organs were isolated after perfusion with 50 ml PBS and quick-frozen in liquid nitrogen.

### Antibodies

We used monoclonal Antibodies against murine CD11b (M1/70), CD86 (GL-1), CD45 (104) from BD Biosciences, CD16/CD32 (2.4G2) from eBioscience for FACS analysis. Antibodies used for the OSC, spinal cord and brain histology: rat-MBP (1:500) from Millipore, rabbit-Iba1 (1:500) from WAKO Chemicals, mouse-Neurofilament (NF-M) (1:1000) from Convance Laboratories Inc., giunea pig-GFAP (1:1000) from SYnaptic Systems, rat-CD68 (1:500) from BioLegend, rat-Mac3 (1:500) from BioLegend, rat-TLR3 (1:500) from BioLegend, rb-pIRF7 (1:400) from Bioss-Antibodies and rabbit-CCR2 (1:500) from Bioss-Antibodies and rtLAMP2 (1:400) von BioLegend. A polyclonal crossreacting anti-GFP antibody was purchased from Abcam. Biotin conjugated donkey-anti-rabbit, as well as normal sera from mouse, rat and donkey were purchased from Jackson Immuno Research. All secondary antibodies conjugated with fluorophores (Cyanine Dye Cy2, Cy3 and Cy5) were purchased from Life Technologies and used in a dilution of 1 to 500.

### Flow cytometry and cell sorting

Co-expression of indicated cell surface markers with YFP expression was analyzed on a FACS Canto II (Becton Dickinson). Cells were pregated as DAPI^-^. Isolated CNS mononuclear cells or primary adult microglia were sorted on a FACS Aria cell sorter (Becton Dickinson) for CD11b and CD45 and YFP reporter allele expression. RNA isolation of *ex vivo* sorted primary microglia was performed with the mirVana miRNA isolation kit (Ambion Inc). RNA isolation of *in vitro* sorted primary adult microglia was performed with RNA isolation kit (Fluka).

### Intracerebroventricular injection

Mice were anaesthetized with isofluran and placed in a stereotactic frame. The skull was exposed and trepanated for injection of 6 μg poly (I:C) (Amersham) into the lateral ventricle. The bregma coordinates were AP: −0.3 mm, ML: +1.0 mm, and DV −3.0 mm.

### Cell culture

For primary adult microglia culture CNS mononuclear cells were isolated from brain and spinal cord of 4–6 week old mice under sterile conditions and cultured in VLE-DMEM (Biochrom) with 10% FCS, 50 μM β-ME and 15% of M-CSF containing supernatant from L929 cells. The protocol was adapted from Ponomarev [21]. Cells were stimulated on d14 with 50 μg/ml poly (I:C), 6 μg/ml CpG2216 (TIB MOLBIOL), 100 ng/ml Lipopolysaccharide (LPS) from *Salmonella minnesota* R595 (List Biological Laboratories, Inc.), 1 μg/ml Pam3CSK4 (Invivogen) or 100 U/ml mouse recombinant IFNβ (R&D Systems) for 6 h or 24 h as indicated or analysis of phagocytosis capacity was performed with DII-coupled myelin isolated according to Norton and Poduslo [22].

Mouse BV2 cells [23,24] were maintained on uncoated petri plates in Dulbecco’s modified Eagle’s medium (DMEM) (Invitrogen) supplemented with Glucose (4.5 g/l), 10% FCS (Invitrogen), 20 mM GlutaMAX (Life Technologies) and penicillin/streptomycin (5 μg/ml) (Life Technologies). Media was changed every 2 days and cells were passaged at a confluence of 80-90% performing trypsinization (Invitrogen).

### Immunofluorescence of microglial cells

Primary microglia or BV2 cells were once washed with PBS, fixed with 4% PFA for 15 minutes and again washed 2 times with PBS. Cells were blocked for 1 h with 5% (v/v) horse serum (Sigma Aldrich) in 0.5% (v/v) Triton X-100 in PBS. Primary antibodies were diluted in 2.5% (v/v) horse serum, 0.25% (v/v) Triton X-100 in PBS and incubated overnight at 4°C. After three times washing for 5 minutes with 0.1% (v/v) Triton X-100 in PBS the cells were incubated with fluorescent secondary antibodies, diluted in 2.5% (v/v) horse serum, 0.25% (v/v) Triton X-100 in PBS, for 1 h at RT. Cells were counterstained with Hoechst (Life Technology) and mounted on glass slides with Immuno Mount (DABCOTM).

### RNA isolation, cDNA synthesis and qRT-PCR

RNA was isolated with RNA isolation Kit (Fluka or Macherey-Nagel). Purified RNA was digested with DNase I (Roche) to remove trace contaminating genomic DNA. An aliquot corresponding to 0.5 – 3 μg of purified RNA was used for first-strand cDNA synthesis using Superscript III reverse transcriptase and oligo (dT) in a final volume of 20 μl according to the manufacturer’s instruction (Invitrogen Life Technologies). cDNA was used for subsequent PCR. Real-time quantification of genes was performed using a SYBR Green RT-PCR assay (Applied Biosystems, USA). Briefly, each 20 μl SYBR green reaction consisted of 5 μl cDNA, 10 μl SYBR Green PCR-mix (2×), 1 μl forward and reverse primer (5pM) and 4 μl distilled water. PCR was performed with the following cycling conditions: 40 cycles of 10 sec at 95°C, 60 sec at 60°C and a separate dissociation step. Specificity of the PCR product was confirmed by examination of the dissociation reaction plots. A distinct single peak indicated only one DNA sequence was amplified during the RT-PCR. The samples were run in duplicates and the level of expression of each gene was compared with the expression of GAPDH. Amplification, detection of specific gene products and quantitative analysis were performed using an “ABI 7500” sequence detection system (Applied Biosystems, USA).

### Organotypic slice cultures

Organotypic slice cultures (OSCs) were generated from 10 days old mice as described before [25]. The cerebellum was cut into 400 μm thick slices using a McIllwain tissue chopper (GaLa Instrumente). OSC were dissociated in ice-cold dissecting medium (Hank’s Balanced Salt Solution (HBSS), Life Technologies) complemented with penicillin/streptomycin (100 U/ml, Life Technologies), 2.5 mg/ml glucose (Sigma Aldrich) and 10 mM kynurenic acid (Sigma Aldrich). OSCs were cultured on Millicell-CM culture plate inserts (Millipore) in culture medium (50% (v/v) MEM, 25% (v/v) HBSS, 25% (v/v) heat-inactivated horse serum, 2 mM glutamine, penicillin/streptomycin (100 U/ml) (all from Life Technologies) and 2.5 mg/ml glucose (Sigma Aldrich) for 3–5 days at 37°C in a humidified atmosphere with 5% CO_2_, and then demyelinated with lysolecithin (0.5 mg/ml, 16 h). After incubation the lysolecithin containing medium was removed and replaced with fresh medium. At this point OSCs were used for all experiments. In some experiments OSCs were treated with 100 U/ml mouse rIFNβ as indicated. For the usage of PLP-EGFP slices, fluorescent images were taken on indicated time points with an Olympus BX51 microscope at low magnification and in sterile conditions.

### Histology

Brain and spinal cord were fixed with periodate-lysine-paraformaldehyde (PLP) overnight, incubated in 10% sucrose followed by 20% and 30% sucrose incubation steps. Organs were frozen in TissueTek (Sakura). Endogenous peroxidase activity and biotin were blocked. The staining of YFP was performed with a polyclonal crossreacting anti-GFP antibody overnight with 0.1% Triton at 4°C. Fluorescence was enhanced with TSA Fluorescein (PerkinElmer) according to the manufacturer‘s instructions. Sections were mounted with DAPI containing Vectashield [26]. Imaging was performed on an epifluorescence microscope (Eclipse TE 2000, Nikon) with digital camera (CCD-1300, Vosskuehler) and overlaid using Adobe Photoshop.

For immunocytochemistry cryo sections were stained with luxol fast blue (solvent blue from Sigma Aldrich) for demyelination and nuclear fast red (Sigma Aldrich) for nuclei. Imaging was performed on a fluorescent microscope (Olympus BX51) with a digital camera (Olympus F-View II).

OSCs were washed two times in warm PBS, fixed 40 minutes in 4% paraformaldehyde (PFA) and permeabilized for 1 h with 1% (v/v) Triton X-100 (Sigma Aldrich) in PBS [25]. OSCs were blocked for 2 h with 5% (v/v) horse serum (Sigma Aldrich) in 0.5% (v/v) Triton X-100 in PBS. Primary antibodies were diluted in 2.5% (v/v) horse serum, 0.25% (v/v) Triton X-100 in PBS and incubated for two days at 4°C. After three times washing for 15 minutes with 0.1% (v/v) Triton X-100 in PBS the OSCs were incubated with fluorescent secondary antibodies, diluted in 2.5% (v/v) horse serum, 0.25% (v/v) Triton X-100 in PBS, overnight at 4°C. OSCs were counterstained with Hoechst (Life Technology) and mounted on glass slides with Immuno Mount (DABCO™). Primary and secondary antibodies used are described above. Imaging was performed on a laser scanning confocal microscope (Zeiss Axiovert 200 M/LSM 510) with a digital camera (Zeiss Axiocam) and a fluorescent microscope (Olympus BX51) with a digital camera (Olympus F-View II). All images were overlaid and processed with Adobe Photoshop. For better visualization of transplanted BV-2 cells on OSCs the cells were circled using Adobe Photoshop software. For quantifying the MBP staining intensity images were analyzed by ImageJ software.

### Statistical analysis

All values in the figures are shown as indicated (mean ± SEM or mean ± SD). Statistical significance was assessed using Student’s *t* test (*p < 0.05; **p < 0.01; ***p < 0.001).

## Results

### IFNβ is produced primarily by microglia during the effector phase of EAE

**Figure 1:**
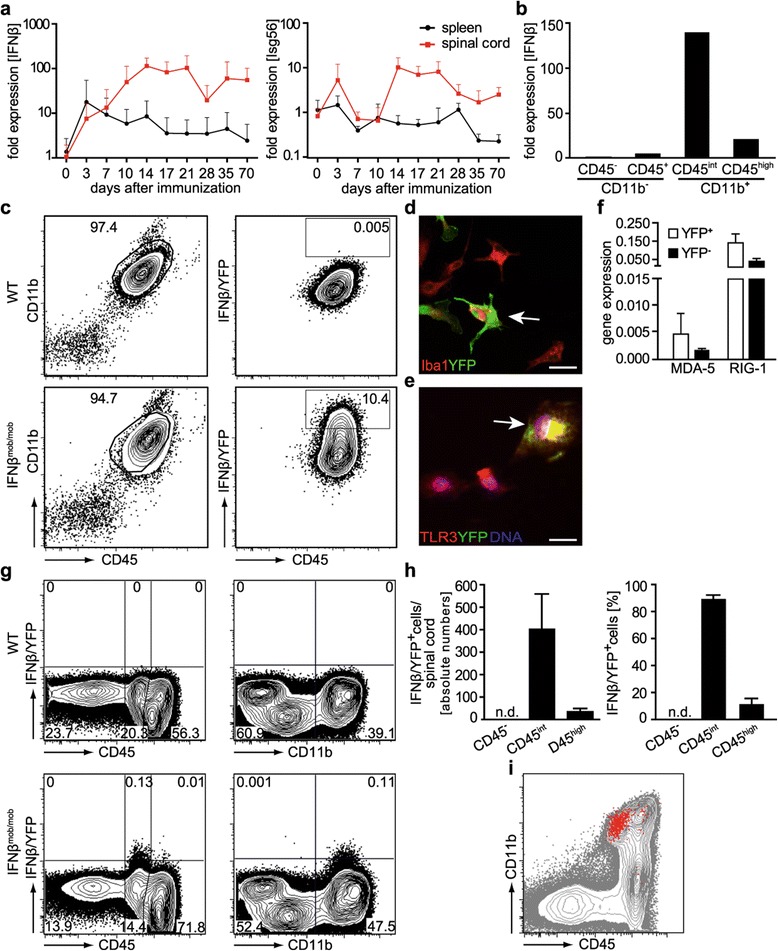
**IFNβ is predominantly produced by microglia during the effector phase of EAE. a** EAE was induced in C57BL/6 N mice by immunization with 200 μg MOG_35–55_ peptide. Pertussis toxin was applied i.p. on d0 and d2. Spleen and spinal cord were isolated at indicated time points after immunization. Relative mRNA expression levels for IFNβ (left) and Isg56 (right) were determined by qRT-PCR. Data are pooled from two independent experiments. n = 6–7. **b** On d17 after EAE induction mononuclear cells from the spinal cord of C57BL/6 N mice were sorted for CD45 and CD11b expression. Relative mRNA expression of IFNβ was determined by qRT-PCR in the indicated cell populations. **c** Primary adult microglia cultures were generated from WT and IFNβ^mob/mob^ mice and stimulated with poly (I:C) for 24 h. IFNβ/YFP expression was analyzed by flow cytometry. **d** and **e** Immunofluorescent stainings of primary adult microglia treated as in **(c)**. Cells were stained with anti-Iba1 **(d)** or anti-TLR3 **(e)** and for YFP for fluorescent microscopy. Scale bar represents 50 μm. **f** Primary adult microglia from IFNβ^mob/mob^ mice were stimulated as in **(c)** and sorted for CD45^+^ CD11b^+^ IFNβ/YFP^+^ vs. CD45^+^ CD11b^+^ IFNβ/YFP^−^ expression. The relative mRNA expression of RIG-I and MDA-5 was determined by qRT-PCR. **g** On d17 after immunization the phenotype of IFNβ/YFP expressing cells from the spinal cord of WT and IFNβ^mob/mob^ mice was determined for CD45 and CD11b by flow cytometry. **h** Quantification of IFNβ/YFP^+^ cells isolated at d17 after immunization from the spinal cord of IFNβ^mob/mob^ mice. Shown is one representative experiment out of 3 independent experiments. **i** Representative dot blot shows an overlay of CD45 and CD11b expression of IFNβ/YFP^+^ (red) and IFNβ/YFP^−^ (grey) cells in the spinal cord of IFNβ^mob/mob^ mice on d17 after immunization. Error bars represent SEM.

To characterize the expression of IFNβ and a representative IFN-inducible gene (Isg56) in CNS autoimmunity over a long term period we immunized C57BL/6 N mice with MOG_35–55_ peptide (hereafter referred to as MOG) and characterized gene expression for 70 days. In the induction phase when clinical symptoms were still absent a significant increase in IFNβ mRNA levels was observed in the spinal cord and the spleen. A similar increase in IFNβ message, however, was also detected in adjuvant only controls. In the CNS, IFNβ mRNA levels increased steadily immediately after MOG immunization and reached a plateau in the effector phase of EAE (around day 14) when clinical symptoms were most severe (Figure 1a, Additional file 1: Figure S1a and S1b). However, during remission of clinical symptoms IFNβ expression were maintained at high levels in the CNS. In the spleen, after a transient increase in the induction phase, IFNβ levels remained low throughout the course of disease (Figure 1a, Additional file 1: Figure S1a). Overall Isg56 expression levels paralleled IFNβ expression levels in the CNS. In the spleen, no significant induction of Isg56 expression could be observed indicating that the functional effects of IFNβ were mainly found in the CNS by its direct activation of CNS resident cells or of circulating cells infiltrating the CNS (Figure 1a). To determine the cellular source of IFNβ we sorted mononuclear cells isolated from the spinal cord of MOG-immunized C57BL/6 N mice at the peak phase of EAE according to CD45 and CD11b expression. The vast majority of IFNβ mRNA was found in the CD45^int^ CD11b^+^ cell fraction comprising mainly microglia. Low levels of IFNβ mRNA were also detected in CD45^high^ CD11b^+^ immigrating myeloid cells. In CD45^+^ CD11b^−^ lymphocytes IFNβ expression remained near the detection limit (Figure 1b). A similar expression pattern of IFNβ mRNA was identified in mononuclear cell subsets from the brain of MOG-immunized mice, although at lower mRNA levels (Additional file 1: Figure S1c). To further analyze the intrinsic ability of microglia to produce IFNβ, we cultured primary adult microglia from wildtype (WT) and IFNβ/YFP knock-in reporter (IFNβ^mob/mob^) mice [17] and stimulated them with the pathogen associated molecular compounds poly (I:C), CpG2216, LPS and Pam3CSK4 (Additional file 2: Figure S2). From these tested stimuli only poly (I:C) induced significant IFNβ expression. Up to 10% of IFNβ^mob/mob^ microglia exhibited IFNβ/YFP expression (Figure 1c, Additional file 2: Figure S2a). Quantitative RT-PCR analyses of FACS-sorted IFNβ-producing primary adult microglia demonstrated only slightly increased MDA-5 and RIG-I mRNA levels when compared to IFNβ non-producers indicating gene expression for intracellular nucleic acid sensing pathways in microglia in general (Figure 1f). Immunofluorescence analysis verified co-expression of the microglial marker Iba1 with TLR3 in IFNβ/YFP^+^ primary microglia cells generated from IFNβ^mob/mob^ mice further demonstrating microglia express pattern recognition receptors for poly (I:C) (Figure 1d and e). We therefore used *in vivo* stimulation with poly (I:C) to initially characterize IFNβ-producing cells in the CNS in response to a strong and local IFNβ inducing stimulus. For this, intrathecal stereotactic microinjection of poly (I:C) was performed on IFNβ^mob/mob^ mice. Here, CD45^int^ CD11b^+^ cells representing microglia were identified as the main producers of IFNβ/YFP in the CNS by flow cytometry (Additional file 3: Figure S3a and S3b). In immunohistology IFNβ/YFP expressing cells showed a microglia-like morphology and localized in periventricular CNS regions (Additional file 3: Figure S3c). Next, we determined the identity of IFNβ/YFP expressing cells in CNS autoimmunity. No significant differences were observed in the disease course of IFNβ^mob/mob^ vs. WT mice (data not shown). Flow cytometric analysis of mononuclear cells isolated from the spinal cord and brain of IFNβ^mob/mob^ mice at the peak of disease (17 days after MOG-immunization) showed intermediate CD45 and high CD11b expression on more than 80% of IFNβ/YFP^+^ cells (Figure 1g–i, Additional file 4: Figure S4). Thus, the majority of endogenous IFNβ producing cells in the CNS during EAE were classified as microglia. Quantitative analysis revealed that IFNβ/YFP expression is restricted to a total of about 400 cells within the spinal cord and less than 50 cells within the brain of MOG-immunized mice (Figure 1h, Additional file 4: Figure S4c). No IFNβ/YFP expression was detectable in mononuclear cells from the spinal cord of naïve IFNβ^mob/mob^ mice (Additional file 4: Figure S4d and S4e). These findings demonstrate that during the EAE effector phase IFNβ is expressed in the CNS primarily by a low frequent subpopulation of microglia.

### IFNβ/YFP producing cells are located within active lesions in the CNS at the peak of disease

**Figure 2:**
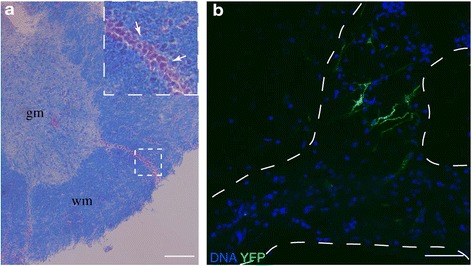
**IFNβ/YFP producing cells localize to active lesions in the CNS at the peak of disease. a** On day 17 after EAE induction spinal cord was isolated from IFNβ^mob/mob^ mice. Histological analysis of lesions in the spinal cord of IFNβ^mob/mob^ mice at peak disease. Spinal cord sections were stained with luxol fast blue for the detection of demyelination and nuclear fast red for visualization of cellular infiltrates. Arrows indicate cellular infiltrates. gm: grey matter. wm: white matter. Scale bar represents 200 μm. **b** IFNβ/YFP producing cells localize to lesions in the spinal cord of MOG-immunized IFNβ^mob/mob^ mice at d17. IFNβ/YFP^+^ cells were stained with a YFP-crossreacting anti-GFP antibody. Dashed lines mark the area of cell infiltrates indicating lesions. Scale bar represents 50 μm.

To characterize the anatomical localization of IFNβ producing cells within the CNS during autoimmunity we performed histological analyses of the spinal cord of IFNβ^mob/mob^ mice 17 days after MOG immunization. At this time point, active lesions could be observed within the grey but more prominently within the white matter of the spinal cord (Figure 2a). Nuclear fast red-positive cell infiltrates were identified within demyelinated CNS sites as determined by luxol fast blue staining (Figure 2a). To identify IFNβ producing cells *in situ* spinal cord sections of IFNβ^mob/mob^ mice were stained for YFP and analyzed by confocal microscopy. IFNβ/YFP signals in cellular bodies and their processes were predominantly detected in spinal cord areas containing high numbers of infiltrating cells underscoring that IFNβ-producing cells accumulate in active lesions of the CNS during EAE (Figure 2b).

### Microglia but not astrocytes or neurons are the source of IFNβ production *in situ*

**Figure 3:**
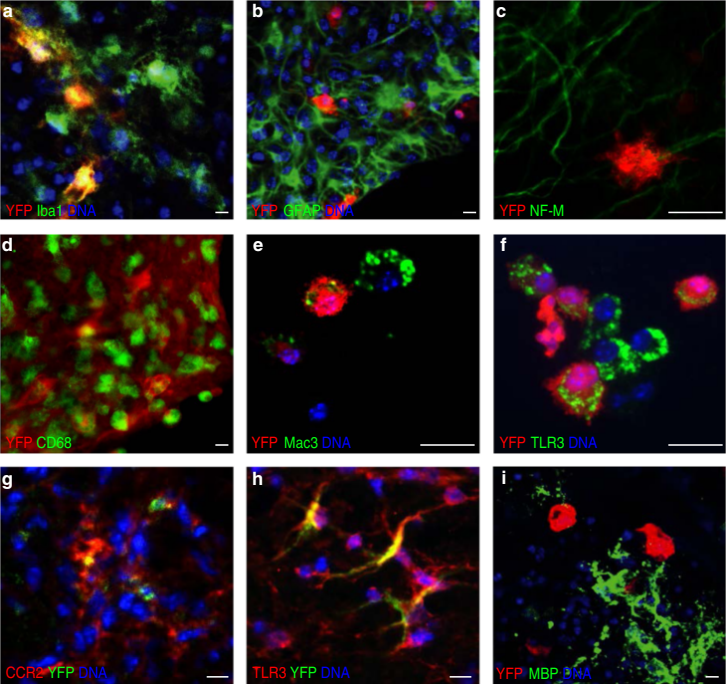
**Microglia but not astrocytes or neurons are the primary source of IFNβ production** ***in situ*** **. a-f** Immunofluorescent analysis of organotypic cerebellar slice cultures (OSCs) for co-expression of IFNβ/YFP and different cell markers. OSCs were prepared as described in the methods and stimulated with 50 μg/ml poly (I:C) for 24 h. OSCs were stained for Iba1 **(a)**, GFAP **(b)**, NF-M **(c)**, CD68 **(d)**, Mac3 **(e)** and TLR3 **(f)** together with IFNβ/YFP. Scale bars represent 25 μm. **g** and **h** EAE was induced by immunization with 200 μg MOG_35–55_ peptide in IFNβ^mob/mob^ mice. Animals with a clinical score >2 were sacrificed at d17 and processed for histological analysis. Shown are representative spinal cord lesions stained for CCR2 **(g)** and TLR3 **(h)**. Scale bars represent 25 μm. **i** Immunofluorescent analysis shows IFNβ/YFP-expressing microglia in close association with myelin debris enriched areas in OSCs. OSCs were prepared and stimulated as described above and stained for MBP and IFNβ/YFP. Scale bar represents 25 μm.

Next, we determined whether non-hematopoietic CNS cells, not detectable in the flow cytometric analyses, contribute to IFNβ production during the inflammatory demyelinating response of the CNS. For this, histological analyses of cerebellar organotypic slice cultures (OSCs) harvested from 10 days old IFNβ^mob/mob^ mice were performed. OSCs were stimulated with poly (I:C) to induce IFNβ production and stained for the microglia marker Iba1, the astrocyte marker glial fibrillary acidic protein (GFAP) and the neuronal marker neurofilament-medium (NF-M). IFNβ/YFP immunoreactivity co-localized exclusively with Iba1 but not with GFAP or NF-M indicating that primarily microglia expressed IFNβ during immune activation *in situ* (Figure 3a-c). IFNβ/YFP positive cells further co-expressed the phagocytic marker CD68 (Figure 3d) and the microglia activation marker Mac3 (Figure 3e) as well as the pattern recognition receptor TLR3 (Figure 3f). Histological analysis of spinal cord lesions from EAE-induced IFNβ^mob/mob^ mice at peak of disease revealed that only a small subset of IFNβ/YFP^+^ cells co-stained positive for CCR2, while the majority of IFNβ-expressing cells was negative for this macrophage/myeloid marker (Figure 3g). Finally, underscoring their capacity to directly respond to TLR3 ligands most IFNβ/YFP expressing cells were found positive for TLR3 (Figure 3h). Using confocal microscopy we found that IFNβ/YFP^+^ cells aggregated in close proximity to areas with a high content of myelin debris caused by spontaneous demyelination due to mechanical stress during cerebellar slice preparation (Figure 3i). These results point towards a possible functional role of IFNβ/YFP producing cells within demyelinated sites in the CNS.

### IFNβ enhances microglia association with myelin debris and their phagocytic activity

**Figure 4:**
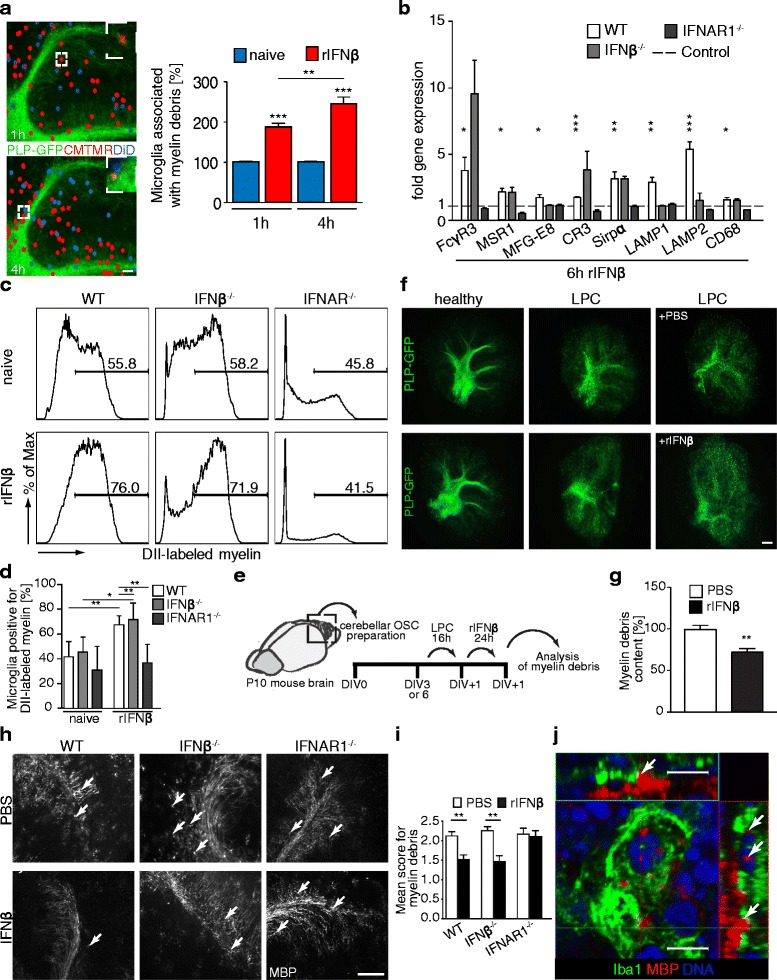
**IFNβ enhances microglia association with myelin debris and phagocytotic activity. a** Co-cultures of sorted primary adult microglia with demyelinated OSCs. Microglia from C57BL/6 N mice treated with rIFNβ and labeled with CMTMR (red) and untreated DiD-labeled (blue) microglia were mixed 1:1 and co-transferred onto LPC-demyelinated OSCs (n = 16) from PLP-EGFP mice. For better visualization transplanted cells were marked using Adobe Photoshop software. Diagrams show percentages of rIFNβ-treated (red) and control (blue) microglia associated with myelin debris. **b** Expression of phagocytosis associated genes in WT, IFNβ^−/−^ and IFNAR1^−/−^ primary adult microglia after stimulation with rIFNβ (6 h). n = 2-4. **c** WT, IFNβ^−/−^ and IFNAR1^−/−^ primary adult microglia were left untreated or stimulated with rIFNβ (24 h). DII-labeled myelin was added for the last hour of stimulation. Uptake of myelin was quantified by flow cytometry. **d** Quantification of phagocytosis activity measured in **(c)** (n = 4–7). **e** Experimental setup for analysis of myelin phagocytosis in OSCs **(f,g)**. OSCs were LPC-demyelinated, treated with rIFNβ or PBS (24 h), and analysed for myelin debris content. DIV, Days *In Vitro*. **f** OSCs from PLP-EGFP mice treated as in **(e)** were imaged. **g** PLP-EGFP-intensity was analyzed by *ImageJ software*. (n = 16–22). **h** OSCs from WT, IFNβ^−/−^ and IFNAR1^−/−^ mice, cultured for 3 days before LPC-demyelination were treated with rIFNβ (24 h) or left untreated and stained for MBP. **i** Quantification of myelin debris in OSCs from WT, IFNβ^−/−^ and IFNAR1^−/−^ mice after rIFNβ treatment. Scoring as described in Additional file 6: Figure S6. (n = 3-4). **j** Confocal Z-stack image from a phagocytic microglia in an WT OSC after poly (I:C) treatment and demyelination. Arrows indicate internalized myelin. Error bars represent SEM **(a,b,g,i)** and SD **(d)**. Scale bars represent 10 μm **(j)**, 100 μm **(a,h)**, 1 mm **(f)**.

Phagocytosis by microglia is a cellular process to clear myelin debris in CNS autoimmunity [27]. To investigate whether IFNβ affected microglia association with myelin debris and phagocytotic capacity we utilized an *ex vivo* transplantation approach of IFNβ treated microglia onto demyelinated OSCs. To this aim microglia were treated with recombinant IFNβ (rIFNβ), CMTMR labeled and co-transferred together with untreated DiD labeled cells at a 1:1 ratio onto demyelinated OSCs prepared from PLP-EGFP mice [20,28]. Thus, migration of IFNβ-stimulated versus untreated control microglia could be monitored on a single viable OSC together with GFP-tagged myelin debris structures. Demyelination occurred broadly on the OSC after the lysophosphatidylcholin (LPC) treatment, without harming the neuronal structures (Additional file 5: Figure S5a). Quantification of microglial cells that localized within myelin debris containing areas 1 h and 4 h after transplantation revealed a significantly increased accumulation of IFNβ treated microglia at these sites (Figure 4a). This specific migratory pattern was also observed in BV2 microglia cells upon treatment with IFNβ (Additional file 5: Figure S5b and S5c). These data indicate that IFNβ promotes the localization of microglia to demyelinated CNS sites.

Next, we examined whether IFNβ activates the phagocytic machinery in microglia cells. For this, expression of surface receptors and intracellular proteins involved in phagocytosis and phagosome maturation were assessed in WT, IFNβ^−/−^ or IFNAR1^−/−^ primary microglia as well as the microglia derived, immortalized cell line BV2. Multiple phagocytosis associated genes were markedly upregulated in WT microglia in response to IFNβ (Figure 4b, Additional file 5: Figure S5d and S5e), among those, the sensor molecules complement receptor 3 (CR3), signal-regulatory protein alpha (SIRPα) and Fc gamma Receptor 3 (FcγR3), shown to be directly involved in myelin phagocytosis in microglia and macrophages [29,30]. In addition, IFNβ upregulated microglial expression of molecules functionally involved in the general phagocytic machinery like macrophage scavenger receptor 1 (MSR1), CD68 and milk fat globule-EGF factor 8 (Mfg-E8) [31,32]. Furthermore, lysosome-associated membrane protein (LAMP) 1 and LAMP2 expression, both important for phagosome assembly were enhanced in response to IFNβ [33]. MBP positive vesicular structures could be observed within IFNβ-activated, LAMP2^+^ primary microglia indicating active phagocytosis (Additional file 5: Figure S5f). The fact that most phagocytosis associated genes and Isg56 were also upregulated in IFNβ^−/−^ microglia indicated a limited contribution of endogenously produced IFNβ to this gene activation (Figure 4b, Additional file 5: Figure S5d and S5g). We also verified the induction of Isg56 gene expression in WT and IFNβ^−/−^ microglia (Additional file 5: Figure S5g). None of these genes were found to be upregulated in IFNAR1^−/−^ microglia verifying the specific effect of IFNβ (Figure 4b).

We further studied whether IFNβ affected the capacity of microglia to specifically phagocytose myelin debris. For this microglia cultures from adult WT, IFNβ^−/−^ or IFNAR1^−/−^ mice and BV2 cells were treated with recombinant IFNβ (rIFNβ) or left untreated and incubated with DII-fluorescent labeled myelin debris for measurement of phagocytosis efficiency by flow cytometry. WT and IFNβ^−/−^ microglia as well as BV2 microglia phagocytosed myelin debris more efficiently following treatment with IFNβ (Figure 4c and d, Additional file 5: Figure S5h and S5i). In contrast, IFNAR1^−/−^ microglia did not exhibit increased phagocytotic rates of myelin debris (Figure 4c and d). These data indicate that IFNβ and IFNAR1-mediated signaling is required to stimulate microglial phagocytosis of myelin debris.

To investigate whether IFNβ would also induce phagocytosis in the *ex vivo* cerebellar slice culture model we used the PLP-EGFP reporter mouse model to monitor the clearance of myelin debris *in situ* [20]. Thus, we induced demyelination in OSCs from PLP-GFP mice with LPC and treated these cultures with rIFNβ (Figure 4e). By using PLP-EGFP OSCs it was possible to measure the myelin content in the same living slice culture before and after the treatment. The myelin debris content was significantly decreased by rIFNβ as visualized by reduced EGFP-PLP signal in slices after 24 h treatment as compared to the PBS-treated demyelinated control cultures (Figure 4f and g). To verify the IFNβ specific effects in this model we prepared OSCs from WT, IFNβ^−/−^ and IFNAR1^−/−^ mice, induced demyelination with LPC and stimulated them with rIFNβ (Figure 4e). The myelin debris pattern was scored using an earlier established method (Additional file 6: Figure S6) [34]. This revealed that WT and IFNβ^−/−^ slice cultures stimulated by rIFNβ showed significantly less myelin debris than untreated OSCs (Figure 4h and i). IFNAR1^−/−^ OSCs exhibited no increase in the removal of myelin debris in response to rIFNβ, providing additional evidence that IFNβ-dependent mechanisms drive myelin debris removal. In a complementary approach we investigated whether endogenously produced IFNβ also affects removal of myelin debris in the CNS. Here OSCs from WT and IFNβ^−/−^ mice were treated with poly (I:C) and subjected to LPC-induced demyelination. We found a significantly higher intensity of myelin debris in OSCs of IFNβ^−/−^ compared to WT slices which further underscored that IFNβ is functionally involved in the clearance of myelin debris (Additional file 5: Figure S5j and S5k). Myelin debris was detected within the cytoplasm of Iba1^+^ microglia cells in LPC-treated OSCs by confocal microscopy (Figure 4j). This points to an uptake of myelin debris by microglia induced by IFNβ.

### Myelin debris accumulation in the CNS is increased in EAE in the absence of IFNβ or its receptor

**Figure 5:**
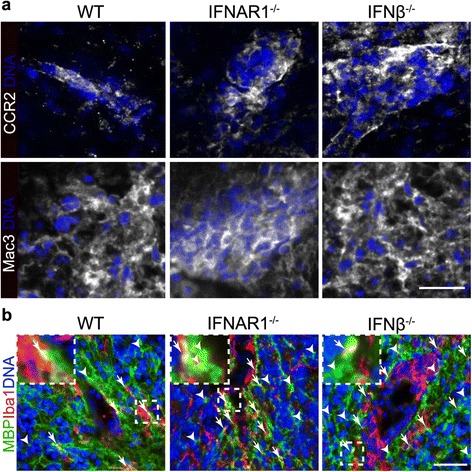
**Myelin debris accumulation in the CNS is increased in EAE in the absence of IFNβ or its receptor.** EAE was induced by immunization with 200 μg MOG_35–55_ peptide in WT, IFNβ^−/−^ and IFNAR1^−/−^ mice. Animals of each genotype with an equivalent clinical score (>2) were sacrificed at d17. Shown are representative brain sections stained for CCR2 and Mac3 **(a)** and Iba1 and MBP **(b)** together with DAPI using fluorescence microscopy. Arrows mark accumulated myelin debris in close proximity to activated microglia and arrow heads mark myelin debris in the lesion structure. Scale bar represents 25 μm **(a)** and 50 μm **(b)**.

According to our data, IFNβ-producing cells are localized in active lesions of the inflamed CNS and phagocytic potential of primary adult microglia was enhanced after stimulation with rIFNβ *in vitro*. To assess the *in vivo* relevance of these findings, we analyzed brain sections and visualized myelin debris accumulation in MOG-EAE induced mice. The majority of lesion infiltrating cells were positive for CCR2 and Mac3 (Figure 5a). To identify the distribution of microglia in active cerebellar lesions and to investigate the myelin debris content we stained brain sections of WT, IFNβ^−/−^ or IFNAR1^−/−^ mice for Iba1 and MBP at peak disease (Figure 5b). As assessed by fluorescence microscopy activated microglia characterized by their amoeboid morphology were present in active lesions in close proximity with granular myelin debris. Enhanced amounts of granular myelin debris were found in lesions of IFNβ^−/−^ and IFNAR1^−/−^ as compared to WT mice where myelin appears more evenly distributed. These results indicate that a lack of IFNβ production or the type I IFN signaling pathway leads to increased myelin debris amounts in active lesions of CNS tissue.

### IFNβ-producing microglia act as orchestrators of myelin debris removal

**Figure 6:**
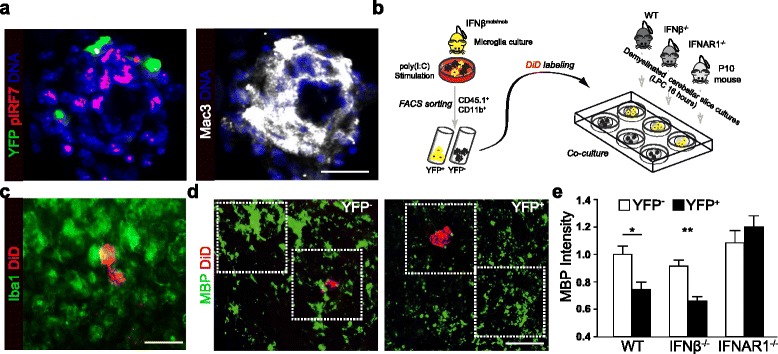
**IFNβ-producing microglia act as orchestrators of myelin debris removal. a** Representative immunofluorescent stainings of primary adult microglia prepared as described above. Cells were co-stained for YFP, pIRF7, and Mac3 together with DAPI. Mac3 and pIRF7 were depicted on separate images for better visualization. Scale bar represents 50 μm. **b** Scheme of the experimental setup. Primary microglia cells were isolated from IFNβ^mob/mob^ mice and treated with poly (I:C) for 24 h. Stimulated microglia (CD11b and CD45) were sorted by flow cytometry for YFP and labeled with DiD. For co-culture experiments YFP-positive and YFP-negative sorted microglia cells were transplanted onto demyelinated cerebellar slice cultures isolated from WT, IFNβ^−/−^ and IFNAR1^−/−^ mice. Demyelination was performed one day prior of transplantation with LPC. **c** Representative image of transplanted DiD labeled microglia in close vicinity to endogenous microglia in the OSC. Scale bar represents 50 μm. **d** Representative images of YFP-negative (left) and YFP-positive (right) sorted microglia after transplantation onto demyelinated OSCs. Scale bar represents 50 μm. **e** Diagram shows intensity of immunostaining for MBP in OSCs from WT, IFNβ^−/−^ and IFNAR1^−/−^ mice transplanted with YFP-negative (white bars) and YFP-positive (black bars) microglia. The quantification of myelin debris in areas surrounding transplanted cells in demyelinated areas was performed by histological analysis of MBP intensity in 0.01 mm^2^ with *ImageJ software* (n = 4 for WT; n = 3 for IFNβ^−/−^ and n = 3 for IFNAR1^−/−^). Data shown as mean + SEM. Statistical significance was determined by Student’s *t* test with *p < 0.05.

Our findings so far indicate that on the one hand microglia produce IFNβ in CNS autoimmunity while on the other hand IFNβ potently induces removal of myelin debris in OSCs as well as CNS lesions in the EAE model. Thus, we wanted to clarify whether microglia additional to their phagocytotic capacity are the relevant orchestrators of IFNβ mediated effects in myelin debris removal *in vivo*. In primary microglia cell cultures we observed that multiple cells positive for phosphorylated IRF7 localize in close proximity to single IFNβ producers in Mac3^+^ cell accumulations indicating promiscuous activation of IFNAR signalling in neighboring cells (Figure 6a). To investigate whether IFNβ-producing microglia harbor the capacity to control clearance of myelin debris in the CNS we transplanted IFNβ-producing or non-producing microglia onto cerebellar slice cultures (Figure 6b). To this end primary microglia cells from adult IFNβ^mob/mob^ mice were stimulated with poly (I:C) and FACS sorted into IFNβ/YFP-producing versus IFNβ/YFP non-producing cells. The separated microglia populations were labeled with DiD and transplanted onto LPC-demyelinated OSCs isolated from WT, IFNβ^−/−^ or IFNAR1^−/−^ mice. DiD transplanted microglia could be visualized embedded within the population of resident microglia of the OSCs (Figure 6c). To quantify myelin debris surrounding transplanted microglia in demyelinated areas histological staining for MBP was performed. As shown in a representative image of slice co-cultures, IFNβ/YFP^+^ microglia integrated in demyelinated regions and associated with affected myelin structures (Figure 6d). Quantitative analysis revealed a significantly decreased intensity of MBP debris in areas proximal to IFNβ/YFP^+^ microglia as compared to IFNβ/YFP^−^ microglia on WT or IFNβ^−/−^ slice cultures (Figure 6e). In contrast, no difference in MBP intensity was measurable between areas proximal to IFNβ/YFP^+^ vs. IFNβ/YFP^−^ microglia transplanted onto IFNAR1^−/−^ slice cultures. Thus IFNβ-expressing microglia can act as inducer cells for clearance of myelin debris. This process is mediated via type I IFN receptor signalling. Taken together, these data demonstrate that IFNβ producing microglia exhibit an enhanced capacity to induce phagocytosis of myelin debris highlighting their functional role as relevant orchestrators for clearance of myelin debris in the CNS.

## Discussion

The high relevance of IFNβ in the therapy of MS and its pleiotropic protective effects in mice and men are irrevocable. Here we identified the so far ill-defined IFNβ producing cells in CNS autoimmunity as primarily microglia in active lesions within the CNS. We further demonstrate that these microglia orchestrate phagocytosis of myelin debris in a process mediated by and dependent on IFNβ.

Our time course analyses showed that IFNβ production and the expression of the IFN-inducible Isg56 in the CNS starts with the onset of clinical symptoms and increases in parallel with the disease score of MOG-induced EAE. These findings extend previous data showing that IFNβ is produced at the peak of EAE exclusively in the CNS [3]. In peripheral lymphoid organs, however, we found IFNβ upregulated early after MOG-immunization and decreased afterwards. This initial expression of IFNβ is also observed when CFA is administered in the absence of MOG and is therefore the result of the immuno-adjuvant containing heat-inactivated *M. tuberculosis* that induces a strong innate immune response. Plasmacytoid dendritic cells have been shown to be responsible for this early produced IFNβ [35] that exacerbates the clinical course of EAE presumably via contributing to the priming of encephalitogenic T cells. Expression of IFNα/β in the effector phase of disease instead was suggested to mediate protective effects by acting directly on myeloid cells [3].

We identified microglia, but not astrocytes or neurons as the major cellular source of IFNβ in the CNS at peak EAE. The identity of IFNβ producing cells in the course of EAE has been a long-standing topic of debate. In general, different cell types, mainly professional antigen presenting cells, are capable of producing IFNβ in the context of immune activation. Plasmacytoid and classical dendritic cells are capable of IFNβ production after TLR9- and TLR3/MDA5-stimulation, respectively [17,36]. During viral infections in the CNS also neurons have been shown to produce IFNβ [37]. *In vitro* murine neurons as well as microglia produced IFNβ after poly (I:C) stimulation [38,39]. Other brain resident cells reported to produce predominantly IFNα are astrocytes as shown in Aicardi-Goutières Syndrome, a rare neurodevelopmental disorder [40]. In MS IFNβ production was detected in active lesions in cells defined as macrophages and astrocytes based on their morphology [41]. Also, based on morphological studies it was suggested earlier that IFNβ may be produced by ramified microglia or infiltrating cells in EAE [4]. Our study defines activated microglia as the prominent IFNβ-producing cell type in the CNS as characterized by an intermediate CD45 and high Iba1 expression as well as a hypermorphic-rounded morphology in the IFNβ/YFP fluorescence reporter mouse model [42-44]. It has been suggested that an activated CD45^high^ CD11b^+^ microglia subset with the capacity to differentiate into macrophages or dendritic-like cells plays an active role in the pathogenesis of EAE [21,45]. However, our data indicate that IFNβ-expressing microglia did not acquire a CD45^high^ CD11b^+^ phenotype during MOG-EAE excluding that they phenotypically and functionally resemble the earlier described subset. The fact that in our study IFNβ production was identified in microglia not only during EAE but also after intrathecal injection of the molecular pathogen compound poly (I:C) points to a specialized function of these CNS resident phagocytes to produce type I IFNs. Of note, at early timepoints after intrathecal poly (I:C) application Khorooshi *et al*. describe a quick mobilization of IFNβ producing myeloid cells from the periphery into the CNS (personal communication). The discrepancies between these two studies could be explained by the different modes intrathecal poly (I:C) application was used (intracerebroventricular vs. intracisterna magna), that may cause induction of divergent chemokine patterns driving leukocyte infiltration. However, in this report, also microglia were shown to contribute to IFNβ production. The IFNβ expression capacity by these cells is in line with our findings on IFNβ production by microglia in CNS autoimmunity without prior poly (I:C) stimulation. We could show that IFNβ-producing microglia localized in the proximity of myelin lesions and exhibit a superior capacity to induce phagocytosis of myelin debris in surrounding cells. It is yet unclear which chemotactic factors guide positioning of microglia into areas of demyelination in CNS autoimmunity. It has been suggested that astrocytes direct migration and activation of microglia and macrophages in demyelinating lesions via expression of CCL2 and CXCL10. Correspondingly microglia and immigrating macrophages in MS lesions stained positive for CXCR3 and CCR2 in MS lesions [46]. Recent elegant data defined CCR2 as a selective marker for infiltrating macrophages in the inflamed CNS [47-49]. Our data do not rule out that immigrating myeloid cells contribute to IFNβ production as indicated by (i) detection of IFNβ mRNA in peripheral tissues early after immunization, (ii) CCR2 expression in a subset of IFNβ/YFP^+^ cells in CNS lesions, and (iii) direct flow cytometric detection of CD45^high^ IFNβ/YFP^+^ cells.

The most important finding of our study is that strategically positioned IFNβ producing microglia within active CNS lesions exhibit a superior capacity to induce myelin debris removal in surrounding tissue phagocytes. Effective clearance of myelin debris is a critical step in the pathogenesis of MS as well as EAE. While microglia have been attributed important roles in the inflammatory response during infections and CNS autoimmunity [50] it is still a matter of debate whether microglia represent efficient phagocytes in the CNS [27]. Here we could show that phagocytosis of myelin debris by microglia was dependent on IFNβ and its receptor IFNAR1. These findings are supported by recent studies showing that *in vitro* microglia deficient in TIR domain containing adapter inducing interferon beta (TRIF)^−/−^ less effectively cleared axonal debris. These microglia further exhibited an increased threshold for activation of interferon-regulated genes, suggesting that IFNβ may upregulate phagocytic activity [51]. In contrast to this, earlier studies suggested that IFNβ suppresses the phagocytosis of myelin debris *in vitro* [3]. In these studies, phagocytic activity, however, was tested in peritoneal macrophages or CD11b^+^ cells in the CNS not differentiating between resident microglia and immigrated macrophages from the periphery. The discrepancy to our data can therefore be explained by differences in the phagocytic activity of microglia and macrophages [52].

The EAE *in vivo* model exhibits a high variability in disease severity and in the localization of CNS lesions between individual animals. Also the suppressive effect of IFNβ and IFNAR mediated signalling on EAE development might reduce demyelination as well as microglia activation [3,4], and Khorooshi et al., personal communication. To eliminate this variability, the unwanted bias and the influence of peripheral immune responses, we used the model of LPC-induced demyelination on OSCs [28] allowing a reproducible and controlled evaluation of IFNβ and microglia mediated effects. IFNβ expressing microglia specifically localized to demyelinated regions in OSCs and further showed lower amounts of myelin debris in their direct proximity in comparison to IFNβ non-producers indicative of a more efficient myelin debris removal. This phagocytosis activating effect was confirmed by transfer of IFNβ/YFP producing microglia on demyelinated WT as well as IFNβ^−/−^ OSCs but was not observed on IFNAR1 deficient OSCs. These data point towards a pivotal function of IFNβ producing microglia in the orchestration of phagocytosis of myelin debris by not only neighboring microglia but also immigrating phagocytes in CNS autoimmunity. It remains to be shown, however, whether the effects observed in EAE can be translated into human MS. While myelin phagocytosis has been suggested to contribute to damage processes in MS by the associated oxidative burst, a number of studies have shown beneficial effects for the effective clearing of myelin debris [27]. Phagocytosis of myelin debris by activated microglia was observed in MS lesions [53] and was essential to promote regeneration [54]. Recently, overexpression of the phagocytosis triggering receptor TREM2 was shown to reduce the severity of clinical symptoms in EAE [55]. Myelin debris was shown to impair remyelination by inhibiting differentiation as well as the recruitment of oligodendrocyte precursor cells after injury [56]. Also, myelin directly inhibited axonal re-growth as it contains several growth inhibitory molecules such as Nogo A [57]. A secondary protective effect of IFNβ-activated myelin phagocytosis might be the induction of a regulatory type of microglia resembling M2 macrophages [58].

## Conclusions

A number of studies indicate that myelin clearance in the CNS after demyelination is protective or ameliorates disease symptoms in EAE and MS. Here, we identify microglia as orchestrators of myelin phagocytosis via production of the protective IFNβ at the peak of CNS autoimmunity. Our findings represent novel insights into the *in vivo* functions of microglia-derived IFNβ and the feasibility of novel therapeutic approaches for MS specifically targeting CNS microglia.

## Additional files

Additional file 1: Figure S1. Clinical score of C57BL/6 N mice after MOG immunization and IFNβ expression in the CNS. a EAE was induced in C57BL/6 N mice by immunization with MOG_35–55_ (200 μg). Pertussis toxin was applied i.p. on d0 and d2. Data represent 6 – 15 animals for each time point. Error bars represent SEM. b C57BL/6 N mice were treated with CFA only. Pertussis toxin was applied i.p. on d0 and d2. The spleen and the spinal cord were isolated at indicated time points after CFA immunization. Relative mRNA expression levels of IFNβ were determined by qRT-PCR. Error bars represent SD. n = 3–4. c IFNβ expression on d17 after MOG-immunization. Shown is IFNβ mRNA expression of sorted cell populations isolated from the brain of C57BL/6 N mice according to their CD45 and CD11b expression. The relative mRNA expression of IFNβ was determined by qRT-PCR. Additional file 2: Figure S2. Induction of IFNβ production in primary adult microglia by molecular pathogen compounds. a and b Primary adult microglia cultures were generated from WT and IFNβ^mob/mob^ mice. On d14 microglial cells were stimulated with 50 μg/ml poly (I:C), 6 μg/ml CpG2216, 100 ng/ml LPS or 1 μg/ml Pam3CSK4 for 24 h. IFNβ/YFP (a) and CD86 (b) expression was analyzed by flow cytometry. Additional file 3: Figure S3. IFNβ expression in the CNS after intrathecal poly (I:C) stimulation. WT and IFNβ^mob/mob^ mice were injected intrathecally with 6 μg poly (I:C). a IFNβ/YFP expression in the brain-isolated mononuclear cells was determined by flow cytometry 24 h after stimulation. DAPI^−^ cells were gated for CD45, CD11b and IFNβ/YFP expression. b Representative dot blot shows an overlay of CD45 and CD11b expression of IFNβ/YFP^+^ (red) and IFNβ/YFP^−^ (grey) cells isolated from the brain of IFNβ^mob/mob^ mice on d17 after immunization. c Localization of IFNβ/YFP expressing cells was determined by immunofluorescence of brain slices of IFNβ^mob/mob^ mice. IFNβ/YFP was stained with a YFP-crossreacting anti-GFP antibody. Nuclei were stained with DAPI (grey) (10-fold magnification). Additional file 4: Figure S4. Microglia express IFNβ/YFP in MOG-EAE in the brain. a EAE was induced in WT and IFNβ^mob/mob^ mice with 200 μg MOG_35–55_ peptide. On d17 after immunization the phenotype of IFNβ/YFP expressing cells from the brain was determined in DAPI^−^ cells stained for CD45 and CD11b by flow cytometry. b Representative dot blot shows an overlay of CD45 and CD11b expression of IFNβ/YFP^+^ (red) and IFNβ/YFP^−^ (grey) cells isolated from the brain of IFNβ^mob/mob^ mice on d17 after immunization. c Quantification of IFNβ/YFP^+^ cells in the brain of IFNβ^mob/mob^ mice on d17 after immunization. Error bars represent SEM. d Mononuclear cells from the spinal cord of naïve WT and IFNβ^mob/mob^ mice were analyzed by flow cytometry as shown in a. e Representative dot blot of an overlay of CD45 and CD11b expression shows no IFNβ/YFP expressing cells under naïve conditions. Additional file 5: Figure S5. Impact of IFNβ on myelin phagocytosis. a Histological analysis of LPC-demyelinated or untreated OSCs stainied with anti-NF-M and anti-MBP antibodies. b Untreated microglia were labeled with DiD (blue circles) and mixed (ratio 1:1) with CFSE labeled cells treated with rIFNβ for 24 h (red circles) before transfer onto LPC-demyelinated OSCs. For better visualization transplanted BV2 cells were marked using Adobe Photoshop software. c Quantification of myelin associated BV2 cells from (b). Diagram shows percentages of IFNβ-treated (red bars) and control (blue bars) BV2 cells associated with myelin debris. (n = 4). d qRT-PCR for genes involved in the phagocytic process in WT, IFNβ^−/−^ and IFNAR1^−/−^ primary adult microglia stimulated with rIFNβ (24 h). (n = 2-4). e Expression of genes involved in the phagocytic process in BV2 microglia upon IFNβ treatment. Cells were stimulated as in (d). (n = 3). f Immunofluorescent staining of primary adult microglia after incubation with DII-labeled myelin for 2 h. Cells were stained with anti-Iba1, anti-LAMP2 and anti-MBP antibodies for fluorescent microscopy. g qRT-PCR analysis of Isg56-gene expression in WT, IFNβ^−/−^ and IFNAR1^−/−^ primary microglia treated with rIFNβ (6 h, 24 h). h and i Uptake of DII-labeled myelin by BV2 cells in response to rIFNβ. BV2 cells were 24 h pre-treated with rIFNβ and incubated with DII-labeled myelin for 1 h. h Quantification of BV2 cells co-localized with DII-myelin. (n = 6). i BV2 cells were stained for Iba1 and Hoechst. j and k Quantitative analysis of myelin debris in demyelinated OSCs of IFNβ^+/+^ and IFNβ^−/−^ mice. Demyelination was induced as in (a) and production of IFNβ induced with poly (I:C). On d6 MBP intensity was analyzed using *ImageJ software*. (n = 3). Error bars represent SEM. Scale bars represent 50 μm (f,i), 100 μm (a,b),1 mm (j). Additional file 6: Figure S6. Scoring table for myelin debris quantification. OSCs were prepared as described and demyelinated by LPC treatment. OSCs were further processed for MBP-staining. The scoring scheme represents the amount of myelin debris in 0.1 mm^2^ in OSCs stained for MBP. A score of “0” points to no visible myelin debris and score “3” displays an area covered with myelin debris (indicated by arrows). Scale bar represents 100 μm.
