# Supplementary figures and images for: IFNβ secreted by microglia mediates clearance of myelin debris in CNS autoimmunity
Source: Acta Neuropathol Commun. 2015 Apr 3;3:20. doi: 10.1186/s40478-015-0192-4 (PMC4383054; doi:10.1186/s40478-015-0192-4)

**a**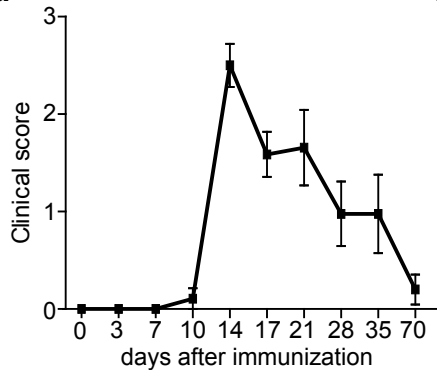**b**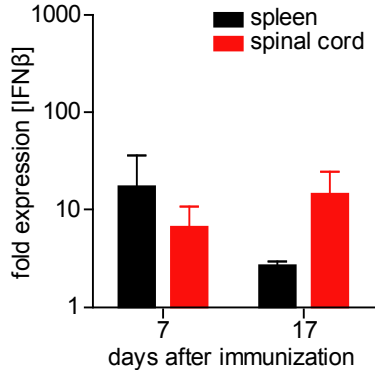**c**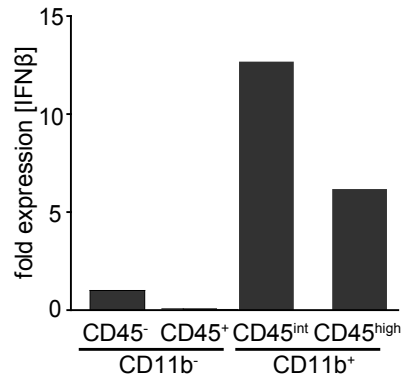

Supplement: Additional file 1: Figure S1. — Clinical score of C57BL/6 N mice after MOG immunization and IFNβ expression in the CNS. a EAE was induced in C57BL/6 N mice by immunization with MOG35–55 (200 μg). Pertussis toxin was applied i.p. on d0 and d2. Data represent 6 – 15 animals for each time point. Error bars represent SEM. b C57BL/6 N mice were treated with CFA only. Pertussis toxin was applied i.p. on d0 and d2. The spleen and the spinal cord were isolated at indicated time points after CFA immunization. Relative mRNA expression levels of IFNβ were determined by qRT-PCR. Error bars represent SD. n = 3–4. c IFNβ expression on d17 after MOG-immunization. Shown is IFNβ mRNA expression of sorted cell populations isolated from the brain of C57BL/6 N mice according to their CD45 and CD11b expression. The relative mRNA expression of IFNβ was determined by qRT-PCR. [file 40478_2015_192_MOESM1_ESM.pdf]

**a**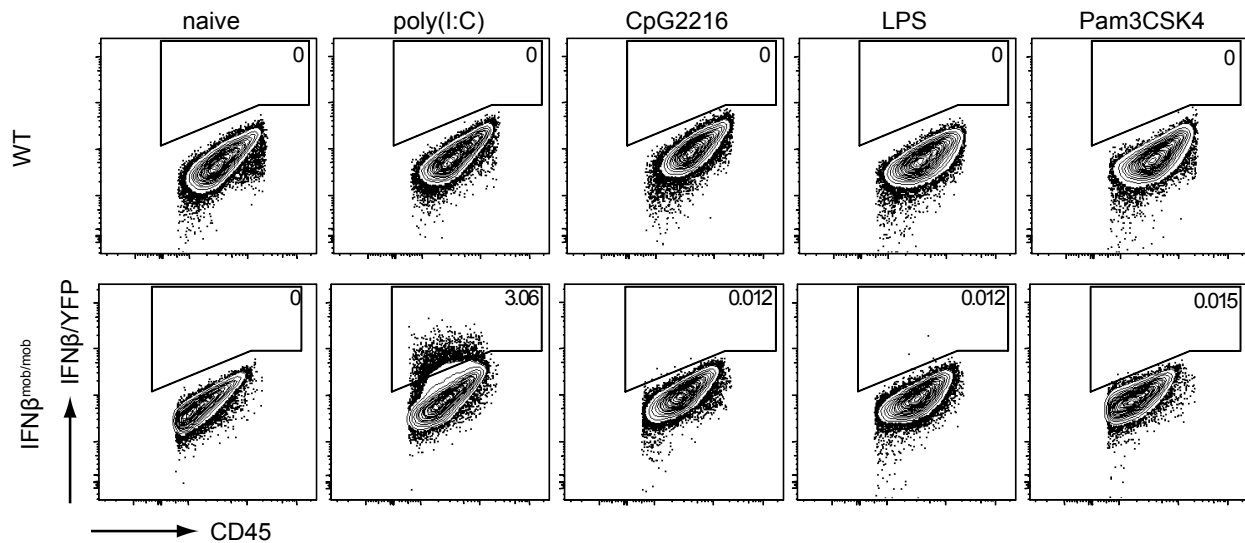**b**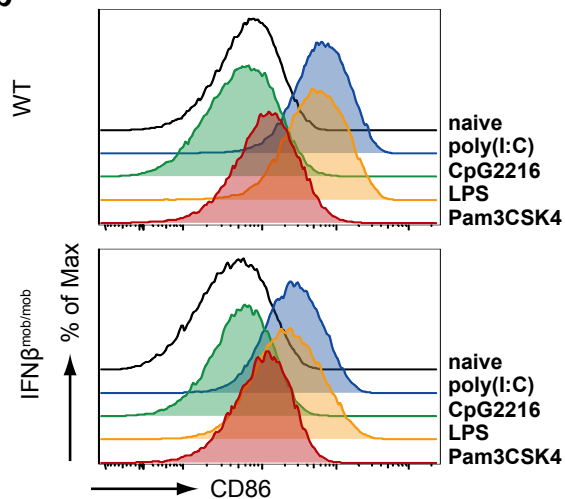

Supplement: Additional file 2: Figure S2. — Induction of IFNβ production in primary adult microglia by molecular pathogen compounds. a and b Primary adult microglia cultures were generated from WT and IFNβmob/mob mice. On d14 microglial cells were stimulated with 50 μg/ml poly (I:C), 6 μg/ml CpG2216, 100 ng/ml LPS or 1 μg/ml Pam3CSK4 for 24 h. IFNβ/YFP (a) and CD86 (b) expression was analyzed by flow cytometry. [file 40478_2015_192_MOESM2_ESM.pdf]

**a**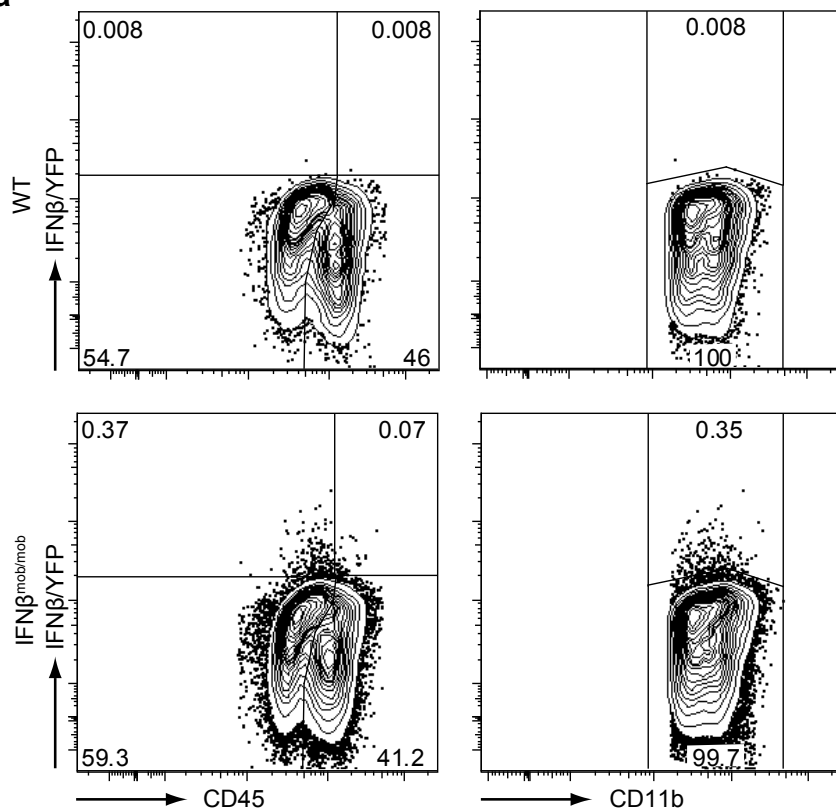**b**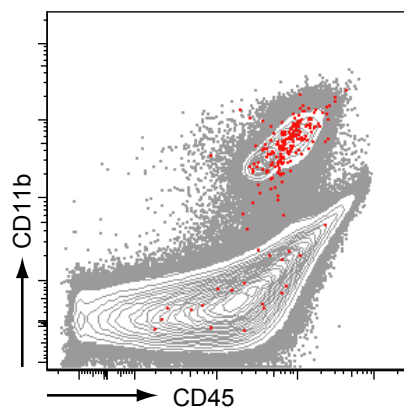**c**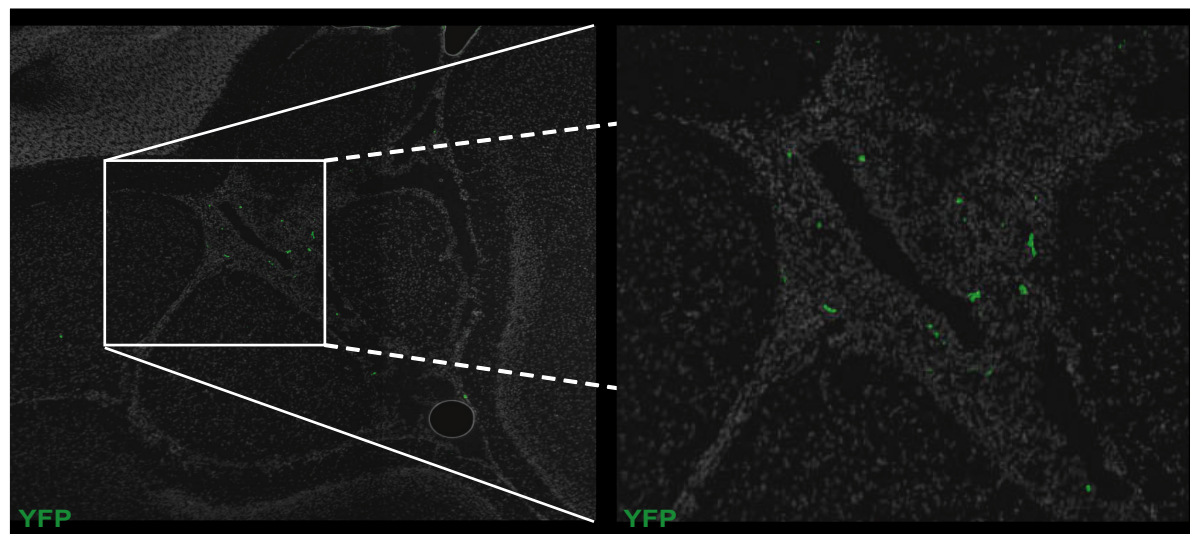

Supplement: Additional file 3: Figure S3. — IFNβ expression in the CNS after intrathecal poly (I:C) stimulation. WT and IFNβmob/mob mice were injected intrathecally with 6 μg poly (I:C). a IFNβ/YFP expression in the brain-isolated mononuclear cells was determined by flow cytometry 24 h after stimulation. DAPI− cells were gated for CD45, CD11b and IFNβ/YFP expression. b Representative dot blot shows an overlay of CD45 and CD11b expression of IFNβ/YFP+ (red) and IFNβ/YFP− (grey) cells isolated from the brain of IFNβmob/mob mice on d17 after immunization. c Localization of IFNβ/YFP expressing cells was determined by immunofluorescence of brain slices of IFNβmob/mob mice. IFNβ/YFP was stained with a YFP-crossreacting anti-GFP antibody. Nuclei were stained with DAPI (grey) (10-fold magnification). [file 40478_2015_192_MOESM3_ESM.pdf]

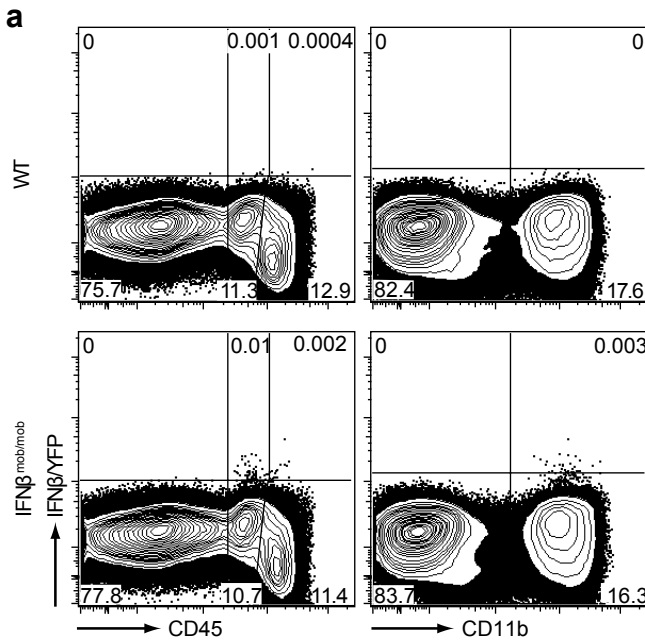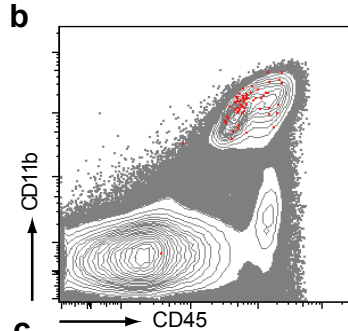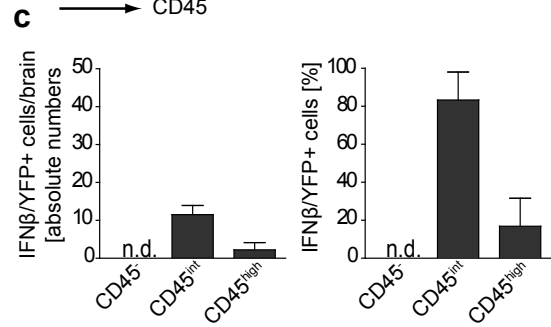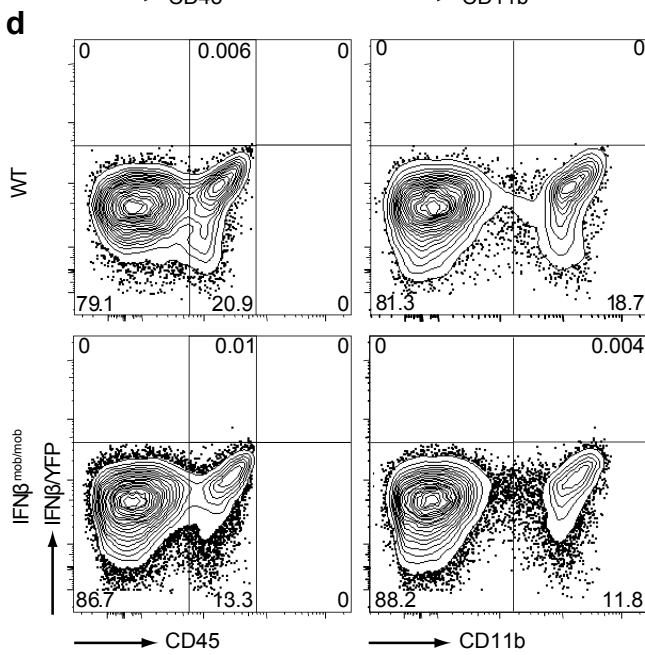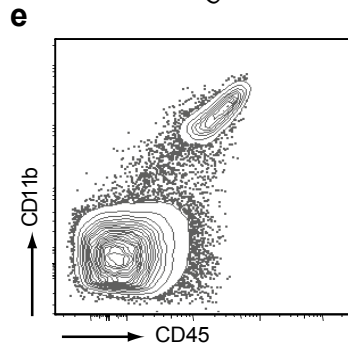

Supplement: Additional file 4: Figure S4. — Microglia express IFNβ/YFP in MOG-EAE in the brain. a EAE was induced in WT and IFNβmob/mob mice with 200 μg MOG35–55 peptide. On d17 after immunization the phenotype of IFNβ/YFP expressing cells from the brain was determined in DAPI− cells stained for CD45 and CD11b by flow cytometry. b Representative dot blot shows an overlay of CD45 and CD11b expression of IFNβ/YFP+ (red) and IFNβ/YFP− (grey) cells isolated from the brain of IFNβmob/mob mice on d17 after immunization. c Quantification of IFNβ/YFP+ cells in the brain of IFNβmob/mob mice on d17 after immunization. Error bars represent SEM. d Mononuclear cells from the spinal cord of naïve WT and IFNβmob/mob mice were analyzed by flow cytometry as shown in a. e Representative dot blot of an overlay of CD45 and CD11b expression shows no IFNβ/YFP expressing cells under naïve conditions. [file 40478_2015_192_MOESM4_ESM.pdf]

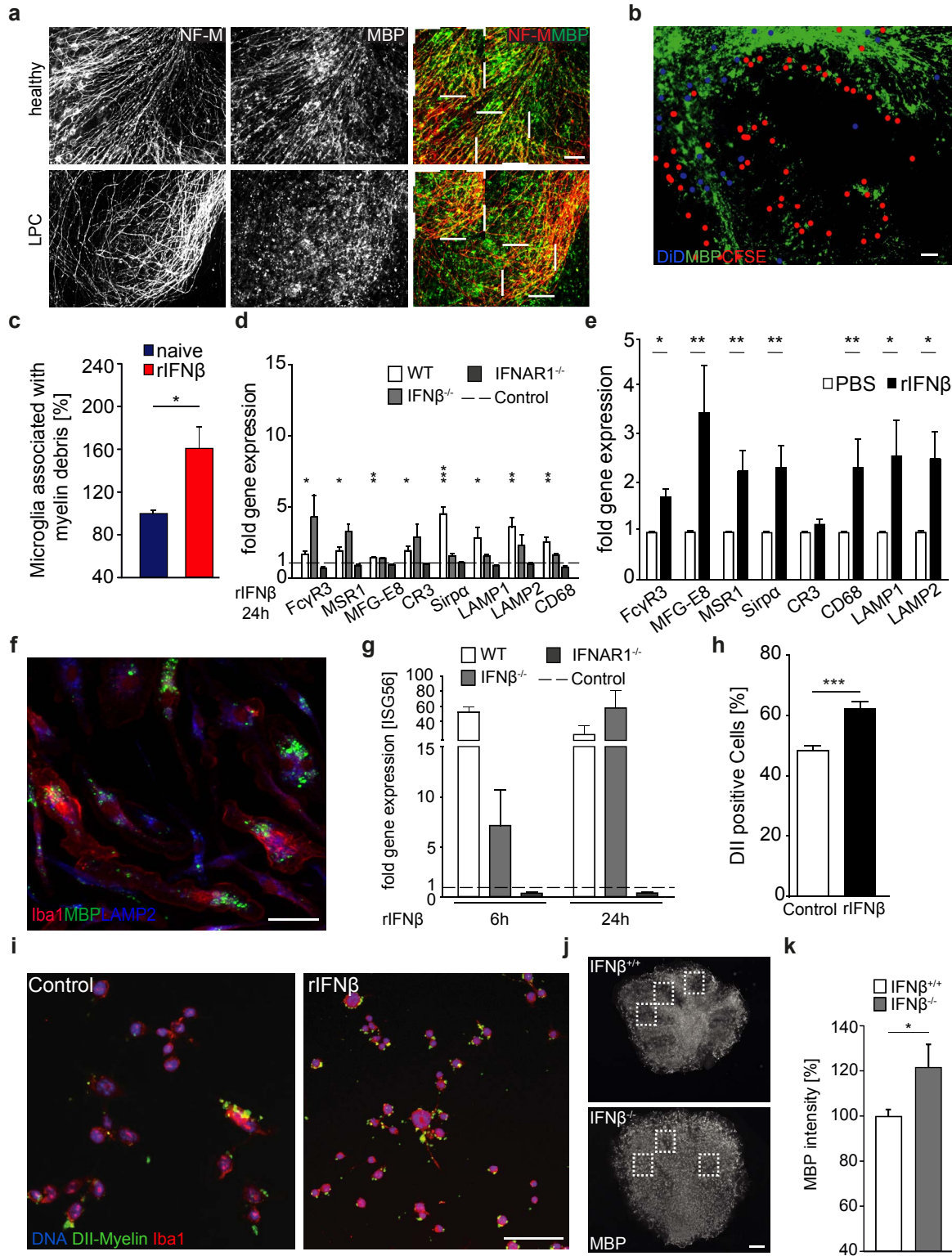

Supplement: Additional file 5: Figure S5. — Impact of IFNβ on myelin phagocytosis. a Histological analysis of LPC-demyelinated or untreated OSCs stainied with anti-NF-M and anti-MBP antibodies. b Untreated microglia were labeled with DiD (blue circles) and mixed (ratio 1:1) with CFSE labeled cells treated with rIFNβ for 24 h (red circles) before transfer onto LPC-demyelinated OSCs. For better visualization transplanted BV2 cells were marked using Adobe Photoshop software. c Quantification of myelin associated BV2 cells from (b). Diagram shows percentages of IFNβ-treated (red bars) and control (blue bars) BV2 cells associated with myelin debris. (n = 4). d qRT-PCR for genes involved in the phagocytic process in WT, IFNβ−/− and IFNAR1−/− primary adult microglia stimulated with rIFNβ (24 h). (n = 2-4). e Expression of genes involved in the phagocytic process in BV2 microglia upon IFNβ treatment. Cells were stimulated as in (d). (n = 3). f Immunofluorescent staining of primary adult microglia after incubation with DII-labeled myelin for 2 h. Cells were stained with anti-Iba1, anti-LAMP2 and anti-MBP antibodies for fluorescent microscopy. g qRT-PCR analysis of Isg56-gene expression in WT, IFNβ−/− and IFNAR1−/− primary microglia treated with rIFNβ (6 h, 24 h). h and i Uptake of DII-labeled myelin by BV2 cells in response to rIFNβ. BV2 cells were 24 h pre-treated with rIFNβ and incubated with DII-labeled myelin for 1 h. h Quantification of BV2 cells co-localized with DII-myelin. (n = 6). i BV2 cells were stained for Iba1 and Hoechst. j and k Quantitative analysis of myelin debris in demyelinated OSCs of IFNβ+/+ and IFNβ−/− mice. Demyelination was induced as in (a) and production of IFNβ induced with poly (I:C). On d6 MBP intensity was analyzed using ImageJ software. (n = 3). Error bars represent SEM. Scale bars represent 50 μm (f,i), 100 μm (a,b),1 mm (j). [file 40478_2015_192_MOESM5_ESM.pdf]

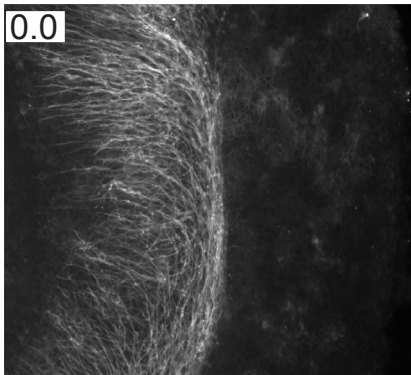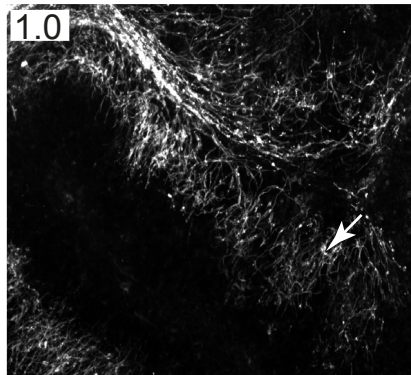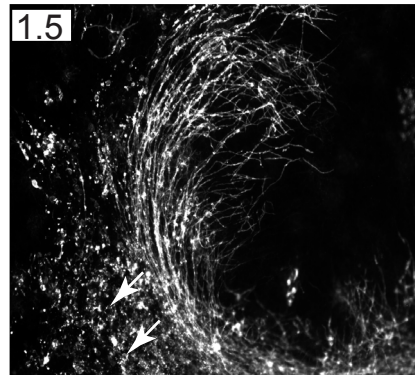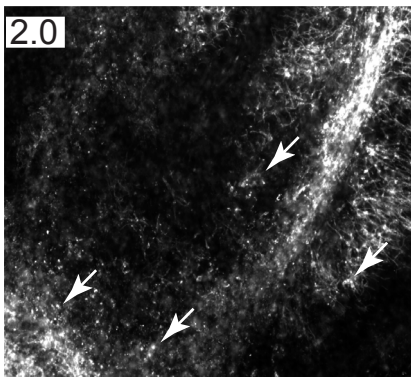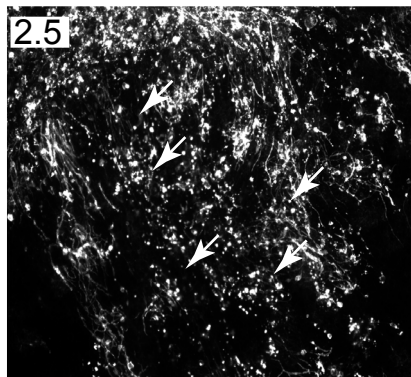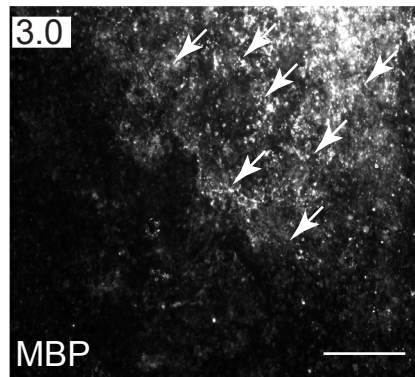

Supplement: Additional file 6: Figure S6. — Scoring table for myelin debris quantification. OSCs were prepared as described and demyelinated by LPC treatment. OSCs were further processed for MBP-staining. The scoring scheme represents the amount of myelin debris in 0.1 mm2 in OSCs stained for MBP. A score of “0” points to no visible myelin debris and score “3” displays an area covered with myelin debris (indicated by arrows). Scale bar represents 100 μm. [file 40478_2015_192_MOESM6_ESM.pdf]
